# Review of "The Twelfth West Coast Retrovirus Meeting" and "The Twenty-third Annual Symposium on Nonhuman Primate Models for AIDS"

**DOI:** 10.1186/1742-6405-3-1

**Published:** 2006-01-11

**Authors:** J Scott Cairns

**Affiliations:** 1 Henry M Jackson Foundation, Mercer Island WA, USA

## Abstract

Two recent meetings held on the west coast of the USA highlighted current work being done in the field of retrovirology and AIDS. The meetings, "The Twelfth West Coast Retrovirus Meeting" (Palm Springs CA; October 6–8, 2005), and the "Twenty-third Annual Symposium on Nonhuman Primate Models for AIDS" (Portland OR; September 21–24) covered a broad range of topics. The highlights covered here are not meant to be inclusive but reflect presentations of interest in the identification and development of new HIV therapies and the role played by animal models in their development.

## New inhibitors

To date, the only commercially available anti-retroviral drugs target three viral proteins, HIV reverse transcriptase, protease, and gp41. In view of the emergence of drug resistance and toxicities associated with the use of current retroviral inhibitors, the need remains for new drugs whose resistance profiles do not overlap with the current arsenal. An obvious way to circumvent this problem is with drugs that target other critical viral proteins. Integrase inhibitors and chemokine receptor inhibitors are currently being tested in late stage clinical trials. While no data on these new inhibitors was presented at these meetings, the maturation inhibitor 3-O-(3', 3'-dimethysuccinyl)-betulinic acid (DSB) was discussed. This drug is currently being tested by Panacos Pharmaceuticals in phase II trials, but its mechanism of action is poorly understood.

C. Aiken (Vanderbilt Univ) reported on his group's efforts to understand the mechanism of action of DSB. It is known that this molecule inhibits HIV replication by delaying the last step in Gag processing, the release of the spacer peptide SP1 from the C-terminus of the capsid protein (CA). The findings reported in Palm Springs, that DSB is incorporated into virus particles, and that mutations in CA that confer DSB resistance also inhibit DSB incorporation into the particle, link the two phenomena. The team also reported an activity of DSB in a cell-free system. To date, this has not been possible, as DSB does not inhibit Gag processing in a cell-free system. Data were presented that DSB added to purified immature HIV-1 cores delayed cleavage of the CA-SP1 junction by HIV protease. To put all these results in a frame work, a model was proposed in which the drug inhibits the final Gag cleavage step by binding to a pocket formed by oligomerization of Gag during particle assembly, thereby blocking access of protease to the CA-SP1 junction. Recently, these results have become online [1].

Other inhibitors of note:

R. Wolkowicz (Stanford Univ) identified several peptides that spared SupT-1 cells from infection with a GFP-expressing HIV-based vector. The mechanism of action of the most intensely studied peptide revealed the involvement of the signalosome as well as the enzyme casein kinase II in HIV-1 infectivity. These early-stage inhibitors are unlikely to make good candidates themselves as inexpensive, orally bio-available drugs of first resort. However, they point the way to additional targets for small molecule drug development.

D Unutmaz (Vanderbilt Univ) demonstrated that TacA, a toxin produced by *Helicobacter pylori*, can suppress HIV infection of primary T cells by a mechanism that takes place after reverse transcription but before 2-LTR circle formation. The molecule also down-regulated IL-2 production, thereby inhibiting cellular proliferation. It is unclear if the effects of this molecule carry over to macrophages or other susceptible target cells or if the antiviral properties of this molecule can be dissociated from its toxic effects. This group also reported on the anti-HIV activity of several amphibian skin-derived antimicrobial peptides. Data from 3 peptides were presented; each inhibited HIV in the low micromolar range. They were generally non-toxic in vitro, but the best of them had an inhibitory effect on T cell proliferation in a concentration range that was not far (2–3 fold) from the HIV-inhibitory range. These results have recently been published [2].

### Host factors as targets for therapy

A number of factors encoded by target cells that have been identified that appear to play critical roles in the HIV-1 infection process. Two of them, TRIM5α and APOBEC3G, which have been recently identified, received particular attention at these meetings. If appropriately targeted, both factors could play critical roles as targets for therapies to inhibit HIV infection.

J Sodroski (Dana-Farber Cancer Institute) summarized studies on TRIM5α a factor identified by his group as playing a critical role in restricting HIV-1 infection in old-world monkeys [3]. The rhesus version of this host cell protein prevents HIV-1 infection of rhesus cells but its mechanism of action is poorly understood. Using an assay that distinguishes alternative forms of capsid in the recently infected cell, this team concluded that TRIM5α does not inhibit uncoating *per se *but rather increases ubiquitination and subsequent disruption of the core of the incoming virus particle by a proteasome-independent pathway. Further, this team identified a region responsible for the markedly different anti-HIV-1 activities of rhesus vs. human TRIM5α. These results have recently been published [4].

Working on the same host cell protein, P Gallay (Scripps Institute) described his group's findings on the mechanism of TRIM5α inhibition of HIV. This group also concluded, on the basis of proteasome inhibitor studies, that TRIM5α-CA interaction results in CA disruption, possibly by enhancing degradation in a proteasome-independent pathway. Further, the TRIM5α-CA interaction does not result in discernable alterations of the pre-integration complex (PIC) but the group speculated that nuclear import of the PIC might nevertheless be affected. Together, the two group's findings give insight into potential new mechanisms for virus inhibitor development.

Although both the Sodroski and Gallay groups reported on the lack of involvement of the proteasome in TRIM5α-mediated CA disruption, T Hope (Northwestern Univ) presented evidence that treatment of TRIM5α-expressing cells with a proteasome inhibitor has a profound effect on the sub-cellular distribution of TRIM5α. Using either a YFP-TRIM5α fusion protein or antibody to simian/human TRIM5α, the investigators found that proteasome inhibition disrupts the normal distribution of TRIM5α in small cytoplasmic bodies, causing an increase in the size and a decrease in the number of these bodies. However, the effect of observed TRIM5α redistribution on HIV-1 replication needs to be established.

As a practical matter relating to TRIM5α mediated HIV-1 restriction in monkeys, B Torbett (Scripps) reported on his group's efforts to improve the transduction efficiency of HIV-1 vectors in non-human primate (NHP) cells. In general, these vectors are very poor at transducing NHP cells due to the ability of rhesus TRIM5α to restrict their expression at a post-entry step (see above). The Torbett and Gallay groups have identified a series of naturally occurring HIV-1 capsid mutants that evade TRIM5α restriction and improve transduction efficiency in several NHP cell lines. Efforts to examine this issue in primary NHP cells are underway.

The gene therapy field, which has had its share of recent setbacks, received an indirect boost from the findings of D Douek (Vaccine Research Center). In general, current gene therapy approaches attempt to modify HIV-1-susceptible cells so that they can resist HIV infection. As such, the approaches are in theory incapable of preventing indirect effects of the virus from damaging the 'protected' cell. There has been much debate over the relative contributions of indirect vs. direct effects of the virus on CD4+ cell depletion. Dr. Douek presented recently published evidence [5] that as much as 60% of mucosal CD4+ cells contained HIV DNA during acute infection. Although no doubt many of these cells are infected non-productively, the high percentage of infected cells suggests that much of the T cell depletion seen in this compartment can be ascribed to the effects of direct infection of these cells. The disparity between measured levels of HIV DNA and RNA suggests that even non-productive infection may be lethal to the target cell.

An interesting pair of talks on APOBEC3G highlighted the rapid pace at which this field has progressed, as well as some areas that are still incompletely understood. W Greene and his associate, J Kreisberg (Gladstone Inst), summarized their findings on the mechanism of action of APOBEC3G. Although it is well-established that one of the enzymatic activities of this factor is its cytidine deaminase activity, which results in G->A hypermutations, sequence analysis of env regions from HIV produced in the presence of APOBEC3G does not substantiate a major role for this enzymatic activity in the HIV inhibitory action of this protein. Dr. Green previously reported on the existence of 2 molecular forms of APOBEC3G, an enzymatically active low molecular weight form and an inactive high molecular weight form [6], and suggested that the cytokine environment in secondary lymphoid organs may contribute to the permissive state of target cells in these organs. He further hypothesized that strategies that convert an inactive high MW form to an active low MW form in susceptible target cells might have promise as HIV inhibitors. Clearly, another approach would be to inhibit the Vif-APOBEC interaction, an avenue that is being pursued by several groups.

N Landau (Salk Institute) summarized his group's findings on the species-specificity of the Vif-APOBEC3G interaction. This interaction is thought to cause the degradation of APOBEC3G, thereby suppressing its HIV inhibitory activity. This team has identified the region in APOBEC3G responsible for this interaction as well as the sites responsible for encapsidation in virions and for its cytidine deaminase activity. In addition, they report on the anti-HIV/SIV activity of different APOBEC family members from primates and mice. A point of difference between the Landau and Greene teams is the importance of the cytidine deaminase activity of this protein, with the Landau group considering this to be an essential activity for HIV inhibition. Resolution of this issue is important as it will determine the potential usefulness of this enzymatic activity as a target for therapy development.

## Viral accessory proteins

A number of presentations that were more heavily focused on basic virology of viral accessory proteins nonetheless had implications for the design of therapies against new targets.

P Cannon (Children's Hospital Los Angeles) reported on her group's studies suggesting that the intracellular pathway by which Vpu traffics to the membrane is critical to its function to enhance virus release. Although this is perhaps not a surprising result, the elegant studies were supported by three separate lines of evidence: 1) a HeLa cell line variant that is defective in its ability to respond to Vpu is also defective in the trafficking of alkaline phosphatase, an enzyme known to be dependent on interaction with the intracellular trafficking protein AP-2; 2) siRNA knockdown of AP-2 in normal HeLa cells disrupts Vpu function; and 3) mutational analysis of the cytoplasmic domain of Vpu suggested a role for an AP-2 interaction motif in Vpu function.

Nef has been the focus of M Powell's group (Morehouse Univ) for a number of years. At the Palm Springs meeting, Dr. Powell focused on the role of Nef in virions, finding that a Nef-cyclophilin A fusion protein can overcome the block to HIVΔnef replication, and that stimulating reverse transcription in viral particles, which is thought to result in partial uncoating of the core, overcomes the block to HIV infection. These findings led the team to speculate that the complex of Nef and cyclophilin A is involved in the uncoating process.

## Latent HIV reservoirs

In addition to the development of viral drug resistance, the problem of viral latency is a clear impediment to the goal of completely eradicating virus from the infected individual. The half-life of the 10^5^-10^7 ^latently infected cells in HAART treated individuals is nearly 4 years [7]. This makes the prospect of lifelong anti-retroviral treatment the only currently available option for treating HIV-1 infection. It would clearly be desirable to speed up the decay of this reservoir, if eradication could be achieved, with the ultimate goal of curing the individual of HIV infection.

D Margolis (Univ North Carolina) discussed his group's findings from a phase I study of valproic acid (VPA). This drug is an inhibitor of histone deacetylase 1, a critical enzyme in chromatin remodeling and a necessary player in maintaining HIV-1 in a latent state in resting CD4 cells. In the study, 4 patients with durably suppressed HIV-1 were treated with VPA and the fusion inhibitor T-20 for 3 months. Three of the 4 patients displayed partial but significant reductions in the frequency of latently infected cells over the time course of the study [8]. Although partial reductions in the size of the latent pool may not be clinically useful, reduction in the size of this pool and increases in its decay rate may make the prospect of eradication more feasible. Thus, although these studies are far from definitive, they point to the need for confirmatory testing in larger scale clinical trials with longer term treatment and longer term follow-up.

J Karn (Case Western Reserve Univ) examined the role of transcriptional factors involved in the re-expression of HIV in latently infected cells. The model described involved an unstimulated Jurkat T cell line containing an integrated lentiviral provirus. In the absence of stimulation, transcription of the provirus in this cell line is limited due to limiting amounts of the transcription factor NF-κB and the DNA helicase, TFIIH. When events were analyzed following cell stimulation, results depended on how the cells were stimulated. When TNFα was the stimulus, RNA polymerase II, NF-κB and TFIIH rapidly (10–30 minutes) accumulated at the promoter, then disappeared, then reappeared between 3–5 hours post-stimulation. In contrast, when the cells were stimulated through their T cell receptor, an initial quick rise in NFAT association with the promoter was followed by a slower but more sustained association of NF-κB with the promoter. The results give a very complicated picture of how HIV transcription might be turned on; mechanisms appear to be stimulus-dependent and can vary depending on the time post stimulation. The relevance of this model to latently infected primary cells is unclear, but unfortunately similar experiments with such cells would be quite difficult, given their scarcity within PBMCs. Implications for therapy development could also be complicated if differences exist in the response of different reservoirs to the different stimuli. This is clearly an area of interest with further research needed to clarify the role of these factors in breaking latency.

## Pathogenic and non-pathogenic models

A number of groups have been looking at the difference between the non-pathogenic SIV infection that occurs in sooty mangabeys (SMs) and African Green Monkeys (AGMs) as opposed to rhesus macaques. By now it has been well documented that, although the virus reaches relatively high set points in SMs and AGMs, markers of immune activation remain much reduced in the non-pathogenic models. These findings were generally confirmed and extended in results presented at the Non-human Primate meeting.

J Milush (Univ Texas SW) reported on 4 SIV infected sooty mangabeys whose CD4+ cells have approached AIDS-defining levels. These unconventional monkeys appear otherwise healthy, however, and 2 of them have had CD4+ cell counts less than 100/ml for more than 3 years. The 2 monkeys that were followed most closely were i.v. inoculated. When CD4+ cell counts were low, circulating virus became dual-tropic (the parent virus was R5 tropic), although the timing of the switch did not appear to account for the depletion. The investigators could find no evidence of immune activation, proliferation or apoptosis as measured by marker expression (K167, CD69, HLA-DR, CD28, CD95, annexin V). Dr. Milush concluded that lack of disease progression in these monkeys is a result of the lack of indirect effects of the virus on CD4+ cells.

P Bostik (Emory Univ) gave an interesting talk on the lack of susceptibility to anergy induction exhibited by central memory CD4+ cells from sooty mangabeys in contrast to Indian rhesus cells. In earlier studies, this group found that alloreactive CD4(+) T cells from humans and rhesus macaques were anergized by TCR-only stimulation whereas alloreactive CD4(+)T cells from SM showed marked proliferation and IL-2 synthesis after restimulation [9] The findings were extended here with the observation that sooty mangabey cells do not up-regulate a gene related to anergy in lymphocytes, GRAIL, following TCR stimulation. While it is unclear why this difference exists between the cells from the two species, it is tempting to speculate on the role this might play in the lack of disease progression in sooty mangabeys.

M Muller-Trutwin (Pasteur Inst) looked at cytokine production in acutely and chronically infected AGMs. These workers documented elevated plasmacytoid dendritic cells in lymph nodes and high levels of circulating IFN-alpha during acute infection. Low Th1 and inflammatory responses were also noted, as has been reported previously by this group [10].

## New models

While the study of various SIV isolates has contributed significantly to our understanding of HIV pathogenesis, there are clear advantages to the study of HIV itself in an animal model. To this end, various SIV-HIV chimeras have been developed over the years but the difficulty has been to develop variants that maintain pathogenicity in animals. Several interesting SHIVs described at the Portland meeting may offer useful additions to our current repertoire of available viruses.

M Ishimatsu (Kyoto) reported on a SHIV encoding HIV protease. The virus has been passaged through monkeys, produces reasonable viral loads, and depletes CD4+ cells so is likely to be pathogenic. The researchers demonstrated that it is sensitive to Kaletra *in vivo *when this protease inhibitor combination was given orally in water. Since it has proven difficult to use protease inhibitors to affect the replication of SIVs in vivo, this variant may prove a useful reagent in the preclinical testing of protease inhibitors in macaques and may also be useful as a model for therapeutic strategies using HAART regimens containing protease inhibitors.

Other SHIVs of interest include ones reported by W Johnson (New England Primate Research Center) in which neutralizing epitopes from HIV env were grafted onto an SIV construct. The two replication-competent viruses they generated may prove a useful resource for analysis of human serum for the presence of particular neutralizing Abs specific for the engrafted epitopes.

L Shek (Aaron Diamond Inst) reported on results with cloned SHIVs in which the only difference between the 2 clones was the env V3 loop. The parent clone was X4 tropic, while the domain-swapped variant used R5. Each clone was used to infect 2 macaques. The R5 clone showed moderate, transient effects on peripheral CD4+ cell counts but induced substantial depletion of intestinal and bone marrow memory CD4+ cells. The X4 clone induced rapid loss of peripheral and lymphoid naïve CD4+ T cells. The findings suggest that co-receptor usage is the major determinant of target cell and tissue specificity *in vivo*, not a surprising finding, but one that is more cleanly addressed using these two cloned isolates that only vary in their V3 loops.

Several talks focused on the use of the low-dose challenge model. This model has recently received focus [11] because it is potentially a better mimic of HIV transmission in humans.

R Otten (Emory Univ) summarized this model in vaccine and published microbicide [11] studies. An addition was the description of studies using oral PMPA given as pre-exposure prophylaxis to prevent transmission. Interestingly, the group found no protection with this regimen, or only a statistically insignificant delay in infection, when PMPA was administered either daily or weekly followed by a rectal low-dose virus challenge. This contrasts with other published studies of post-exposure prophylaxis with PMPA using a high dose challenge [12]. Differences in the challenge dose would seem to be an unlikely explanation for the differences, given the seemingly higher stringency expected with a high-dose challenge. Other differences, including route and timing of delivery of PMPA, or differences in the challenge viruses themselves (the Emory group used SHIV162P3; the Tsai study used SIVmne), may explain these disparate results. However, the findings potentially point to the differences that can be obtained with this model vs. the high dose model. A coordinated effort to compare the two models using the same challenge stock, the same animal species, and the same therapeutic or prophylactic intervention, would clearly be of benefit to the field, especially if the results could be subsequently compared to results in the human.

## Innate immunity

It is becoming increasingly evident that the innate immune system plays a critical role in the control of infectious diseases. Harvesting the potential of this system in the treatment and prevention of HIV is a focus of many groups in academia and industry. R Lehrer described his group's work on theta defensins, peptides that are produced in macaques but not in humans and act as HIV-1 entry inhibitors. In studies reported here, the investigators described in more detail the mechanism by which this small lectin is likely to inhibit virus entry. The molecule appears to have at least 4 carbohydrate binding sites and to exist as trimers. The combined multivalency and oligomeric nature are believed to be responsible for the ability of this and related molecules to bind carbohydrate moieties on envelop molecules from a variety of viruses thereby preventing entry into target cells. The multivalent nature of one theta defensin has recently been published [13]. Due to their peptide nature and their lack of expression in humans, these molecules would make unusual candidates for systemic use unless their likely immunogenicity could be circumvented, but could conceivably find a niche as topical agents.

S Barry (Northwestern Univ) reported on the ability of Langerhans cells (LCs) to present HIV to T cells. These cells, unlike dendritic cells (DCs), have cellular processes that extend to the mucosal surface and thus make likely candidates for the initial encounter of HIV-1 with a susceptible host cell. Unlike DCs, these cells do not express DC-SIGN, but do express Langerin, a cell-surface marker involved in antigen uptake, and CD1a involved in Ag presentation. R5-tropic, but not X4-tropic HIV can infect LC's. In the studies reported here, LC's were found to capture HIV and transfer it to primary CD4+ T cell targets in a process that may involve CD1a, which was clustered in complexes with HIV-1 particles in both LCs and infected CD4+ target cells. The *in vitro *studies were done using an X4 virus, making their relevance to the *in vivo *situation, where R5 viruses are transmitted more efficiently, unclear. Nevertheless the studies point to a role for LCs in transmitting virus without themselves becoming infected.

## Conclusion

The two meetings described here brought together a wide range of investigators focused on understanding HIV-1 and preventing its transmission and pathogenicity. It is clear that we have made much progress in understanding how this virus infects the host and subverts or circumvents cellular mechanisms for its own purposes. However, the fact that today, over 40 million people are infected with the virus, many with little hope of surviving its deadly effects, makes it abundantly clear that we have a long way to go in stopping this scourge. Advances in the discovery and development of new therapies continue to be needed to thwart the ever growing threat posed by the development of drug-resistance as well as the emergence of drug-related toxicities in treated individuals. In addition, the difficulties experienced in supplying treatments in many parts of the world make it imperative that additional ways to stop the infection, including effective vaccines and female-controlled microbicides are urgently needed. The meetings highlighted here offered hope that inhibitors focused on new targets will continue to flow into the pipeline of available treatments and prevention measures while we anxiously await the development of effective vaccines.
